# Which Combinations of Techniques and Modes of Delivery in Internet-Based Interventions Effectively Change Health Behavior? A Meta-Analysis

**DOI:** 10.2196/jmir.4218

**Published:** 2016-06-07

**Authors:** Lenneke van Genugten, Elise Dusseldorp, Thomas Llewelyn Webb, Pepijn van Empelen

**Affiliations:** ^1^ Expertise Group Life Style TNO Leiden Netherlands; ^2^ Department of Public Health Erasmus Medical Center Rotterdam Netherlands; ^3^ Institute of Psychology Leiden University Leiden Netherlands; ^4^ Department of Psychology University of Sheffield Sheffield United Kingdom

**Keywords:** meta-analysis, prevention, health behavior, behavior change, online

## Abstract

**Background:**

Many online interventions designed to promote health behaviors combine multiple behavior change techniques (BCTs), adopt different modes of delivery (MoD) (eg, text messages), and range in how usable they are. Research is therefore needed to examine the impact of these features on the effectiveness of online interventions.

**Objective:**

This study applies Classification and Regression Trees (CART) analysis to meta-analytic data, in order to identify synergistic effects of BCTs, MoDs, and usability factors.

**Methods:**

We analyzed data from Webb et al. This review included effect sizes from 52 online interventions targeting a variety of health behaviors and coded the use of 40 BCTs and 11 MoDs. Our research also developed a taxonomy for coding the usability of interventions. Meta-CART analyses were performed using the BCTs and MoDs as predictors and using treatment success (ie, effect size) as the outcome.

**Results:**

Factors related to usability of the interventions influenced their efficacy. Specifically, subgroup analyses indicated that more efficient interventions (interventions that take little time to understand and use) are more likely to be effective than less efficient interventions. Meta-CART identified one synergistic effect: Interventions that included barrier identification/ problem solving and provided rewards for behavior change reported an average effect size that was smaller (ḡ=0.23, 95% CI 0.08-0.44) than interventions that used other combinations of techniques (ḡ=0.43, 95% CI 0.27-0.59). No synergistic effects were found for MoDs or for MoDs combined with BCTs.

**Conclusions:**

Interventions that take little time to understand and use were more effective than those that require more time. Few specific combinations of BCTs that contribute to the effectiveness of online interventions were found. Furthermore, no synergistic effects between BCTs and MoDs were found, even though MoDs had strong effects when analyzed univariately in the original study.

## Introduction

Online interventions hold great promise for the promotion of health behavior. The Internet is used by many individuals to find health-related information [1]. Three potential advantages of online interventions are high reach, low costs, and convenience for users (eg, timely delivery) [2]. Various meta-analyses have shown that online interventions designed to promote health behavior change can be effective, but that the effectiveness of interventions varies considerably [3-6]. One source of variability is differences in the behavior change techniques (BCTs) that are used by interventions. Research points to the importance of using standard definitions of BCTs [7,8] and has started to identify which BCTs are effective and which are less so. Yet relatively few studies have sought to identify the effectiveness of BCTs in online interventions. One exception is Webb et al [3], who examined the effectiveness of online interventions using a taxonomy of BCTs adapted from Abraham and Michie [9]. Webb et al [3] found, based on univariate analyses, that several BCTs were associated with larger than average effect sizes. Specifically, stress management and general communication skills training had the strongest positive effects, while emotion control training and providing information about others’ approval were not effective.

In addition to deciding which BCTs to use in an intervention, a second challenge for online interventions is how to attract users, encourage them to engage in the intervention and explore the website, and have them return for follow-up visits as necessary [10-12]. This process may be more complicated than traditional intervention methods (eg, a letter, flyer, or video), and it is likely that the usability—or user friendliness—of the intervention [13] has a substantial bearing on the efficacy of that intervention. Usability refers to how easily the features in the intervention are to use and how pleasant it is for the user to engage with the intervention [14]. However, it is unclear which factors influence how usable an intervention is. Therefore, the present meta-analysis aimed to identify factors that influence the usability of interventions, as well as how these factors are related to the effectiveness of interventions.

### Interactions Between Intervention Factors

Webb et al also found that interventions were more effective when more BCTs were included (see also [15]), suggesting that combining BCTs may be more effective than using one or two BCTs in isolation. Indeed, evidence suggests that BCTs can interact and have cumulative (or potentially synergistic) effects. For example, the combination of fear arousal and providing skill information has been shown to be particularly effective in promoting a variety of health behaviors, such as smoking or vaccination [16,17]. Similarly, Michie et al [18] found that interventions that combined self-monitoring with at least one other BCT specified by control theory (eg, goal setting) tended to have larger effects than interventions that used self-monitoring in isolation.

Dusseldorp et al [19] developed and applied a new method for looking at the effectiveness of combinations of moderators (eg, BCTs), which they called meta-CART [20]. Meta-CART combines “Classification And Regression Trees” (CART) and subgroup meta-analysis in such a way that interactions between moderators that can account for variability in effect sizes derived from primary studies can be discovered. Dusseldorp et al found a number of effective combinations of BCTs for promoting physical activity and healthy eating: (1) “Provide information about behavior-health link” with “Prompt intention formation” (mean effect size ḡ=0.46), and (2) “Provide information on consequences” and “Use of follow-up prompts” (*ḡ*=0.44) [19]. However, little is known about synergistic effects in online interventions, which often include several BCTs. This research focuses on BCTs that have a cumulative effect in addition to their univariate positive effect. The second aim of this research is to identify synergistic effects of BCTs (next to their already identified univariate effects) in online interventions aimed at health behavior change.

A third factor that may influence effect sizes is the mode by which the intervention is delivered. Online interventions can differ substantially in their specific modes of delivery (MoD). Webb et al [3] noted that content can be delivered in a more or less interactive manner [18] and that online interventions that employ supplementary delivery modes (notably, text messaging, tailored feedback, access to advisor, telephone, or email) tend to be more effective in univariate analyses [3,10]. However, until now, research has not considered the effectiveness of different combinations of MoDs and how MoDs might interact with the use of particular BCTs and usability factors to determine the efficacy of an intervention. For example, an online peer forum (an MoD) is more likely to provide quick social support (a BCT), and not only at one weekly face-to-face session. Therefore, the third and fourth aims of this research are to identify the most effective combinations of MoDs and the most effective combinations of BCTs, MoDs, and usability factors, respectively. Providing insight into the effects of usability factors and synergistic effects with BCTs and MoDs will also provide a starting point for an evidence-based instrument that can be used to develop new interventions and evaluate the quality and the potential of existing interventions.

### Our Research

This review aims to develop a taxonomy for coding the usability of online interventions and to identify what combinations of BCTs, MoDs, and usability factors influence the effectiveness of online interventions designed to promote health-related behavior. In order to identify these synergistic effects, meta-CART analysis was employed.

## Methods

We considered data from the 85 studies that were included in the meta-analysis of Webb et al [3] for inclusion in this meta-analysis. The studies were published between 1990 and July 2008, in peer-reviewed journals and conference proceedings written in English. This review uses data on the effectiveness of the interventions and the use of BCTs and MoDs. Each intervention was coded by Webb et al for inclusion or exclusion of each of the 40 BCTs from the CALO-RE (Coventry, Aberdeen, and London—Refined) taxonomy of Michie et al [21]. Webb et al also coded 11 modes of delivery used by each of the interventions. Study characteristics were coded by a single author and so information is not available on the reliability of so doing. Evidence suggests that behavior change techniques can be reliably coded [21], but additional research is needed to confirm the extent to which coders can reliably identify modes of delivery and usability.

### Selection of Interventions

Webb et al included studies (1) in which the described intervention was delivered via the Internet, (2) where participants were randomly assigned to conditions, and (3) where health-related behavior was measured after the intervention. This review included interventions that used two or more BCTs, MoDs, or usability factors (if studies included only a single BCT, then it was not possible to study the combined effects of such factors). Second, only effective factors were included in the analyses, because initial meta-CART analyses found that including all factors resulted in a tree without any boosting (or strengthening) effects. A BCT or MoD was considered effective when univariate analyses showed that studies including the BCT or MoD had a higher absolute effect size than the studies not using the specific BCTs or MoD. Because the univariate effects of the usability factors still needed to be investigated, we based our selection only on the effects of the BCTs and MoDs.

A total of 52 studies met the criteria for inclusion in the review. The included studies reported on interventions targeting health-related behavior, such as physical activity (n=13), dietary intake (n=8), and alcohol consumption (n=6). Seven studies addressed multiple behaviors (eg, combined physical activity + dietary intake). The target population varied from children to adults and from the general population to patients at specific risk (eg, patients with diabetes). In order to evaluate the effect of the interventions, all of the studies compared an experimental condition that was exposed to the intervention with an active or passive control condition. The included studies tested the impact of 20 effective BCTs and 7 effective MoDs (see Multimedia Appendix 1 for an overview of all included BCTs and MoDs).

### Taxonomy of Factors That Influence the Usability of an Intervention

Given that studies generally lack a description of the usability of the evaluated intervention, we developed a survey to obtain this information from the original authors. The survey was based on a taxonomy, which described indicators of the usability of online interventions. The existing literature points to various factors that influence how usable an intervention is likely to be, such as guidelines on functionality [22], accessibility [23], usability [24], design [25], user experiences [26], as well as studies of persuasive technology [27] and Shneiderman’s golden rules [28]. First, it was decided which factors were applicable for online interventions targeting health behaviors. Those factors were included in our first draft survey. Second, factors in the draft were grouped and summarized where possible. Third, titles and definitions were adapted to fit the human-computer interaction. Fourth, the factors and definitions were discussed with other interaction specialists. When necessary, factors were adapted or refined and new factors were added.

The result of these steps was a 27-item taxonomy, with 8 subscales: learnability (reflecting how easy it was to accomplish basic tasks the first time that a user encounters the design), efficiency (the speed at which tasks can be performed once users have learned the design), memorability (reflecting how easy it was to re-establish proficiency when a user returns to the design after a period of not using it), errors (likelihood and severity of errors potentially made by users, and how easy it was to recover from such errors), satisfaction (pleasantness of using the intervention), personalization (reflecting the extent to which it was possible to adapt to the intervention to the individual user’s characteristics, preferences, values and self-image), situatedness (the ability of the system to predict and adapt to the user’s dynamic behavior in specifics contexts), and social interaction (reflecting whether the intervention encouraged social interactions). These features and the attributes that compose them are shown in Table 1.

We contacted the 85 authors of the original papers and asked them to rate their intervention using a questionnaire version of our taxonomy and to send us their intervention (if available) so that we could also code features influencing usability. Twelve authors (14%) coded their intervention and six (7%) provided access to their intervention or pictures from the intervention. Calculations and analyses are based on these 12 studies. We calculated scores for each study on each of the 8 scales of the taxonomy by calculating the mean of the items in each scale. Descriptive statistics are used to calculate the scale reliabilities, means, and standard deviations. Scale reliability varied from Cronbach alpha=.69 (efficiency) to .98 (situatedness). The learnability scale had an alpha of only .52 (even after omitting an item) and was therefore not included in the analyses. Scale scores were then dichotomized based on the median (ie, low or high on the scale). Finally, the effect sizes for the low and high group were calculated for each usability factor by using subgroup analyses in Comprehensive Meta-Analysis (CMA) software, version 2.2 [29].

**Table 1:** Usability taxonomy (the intervention referred to in the taxonomy is the intervention itself as well as its embodiment, the user interface).

Usability attributes		Measures (example)
**Learnability**		
	1. Consistency	The intervention is consistent in the use of interface aspects such as layout, buttons, and language (eg, the OK button is always on the left and the Cancel button is always on the right side, consistent use of terminology and look-and-feel)
	2. Conventions	The intervention follows platform conventions (eg, in Windows, the cross at the upper right corner of the screen is always used to close the window)
	3. Intuitiveness	The intervention characteristics intuitively imply its functionality and use (eg, a button with an arrow pointing to the right, implying “go to the next page”)
	4. Visibility of system status	The intervention provides feedback about its (future) state, action, and result (eg, when loading, the system provides a load bar showing how much time has passed and how much time remains)
**Efficiency**		
	5. Flexibility	The intervention caters to a variety of users, both inexperienced and experienced (eg, the system provides both viewable icons, such as a floppy disk, and short-cuts, such as Ctrl-S)
	6. Structure	Using the intervention, users understand the structure of the intervention and know where they are (eg, the intervention provides breadcrumb navigation, ie, showing previous interaction steps and steps to come)
	7. Defaults	The intervention makes use of default settings (eg, fields containing defaults come up selected and the user can replace the default contents with new information—the defaults are user-specific)
**Memorability**		
	8. Tailoring to user group	The intervention speaks the user groups’ language, with words, phrases and concepts familiar to the user group, rather than intervention-oriented terms (eg, intervention contains words and phrases fit for children, intervention uses read-aloud function for low literates)
	9. Recognition rather than recall	The intervention minimizes the user’s effort by making options visible or easily retrievable whenever appropriate (eg, the intervention shows context specific relevant available functionalities instead of referring to a manual where all options are listed)
**Errors**		
	10. Error recovery	The intervention supports undo and redo (eg, the intervention offers a “Go” and a “Back” button)
	11. Error prevention by the system	The intervention prevents problems from occurring and notifies the user if a problem can potentially occur (eg, the intervention indicates which fields are mandatory [*] and applies form validation, such as the right format for postal code)
	12. Error recognition and resolution by the system	The intervention provides error messages expressed in plain language (no codes), which precisely indicate the problem and constructively suggest a solution (eg, when entering a faulty password, the intervention indicates: “Your password is incorrect, please ensure your CAPS LOCK key is off”)
	13. Help by the system	The intervention provides help information that is easy to search and focuses on the user’s task (eg, the intervention has a help function, in the form of a question mark icon, for every text field that needs to be entered)
**Satisfaction**		
	14. Minimalistic	The intervention does not contain elements that are irrelevant or rarely needed (eg, the intervention interface is not cluttered, does not use distracting irrelevant interface elements, and does not require extensive scrolling)
	15. Aesthetic appearance	The intervention is esthetically attractive (eg, includes pictures, colors)
	16. Fun	The intervention is fun to use (eg, the intervention offers a challenge to users and arouses their curiosity)
	17. Modality integrity	The intervention offers information in a suitable modality (eg, information is presented in text when presenting details, information is presented in image when providing an overview)
	18. User control	The user is in control of the intervention (eg, the user initiates actions, the system justifies responses to actions of the user)
**Personalization**		
	19. Adaptability by the user	The user can adapt the intervention to fit to their preferences and skill level (eg, the intervention offers the user to decrease and increase interface’s font size, the intervention offers the possibility to take a tour through the intervention or change the background color)
	20. Adaptiveness to the user	The intervention is aware of the user’s characteristics and adapts the interface to these characteristics (eg, the intervention is aware the user is farsighted and increases the font size)
	21. Adaptiveness to the context	The intervention is aware of the user’s context and adapts the interface to this context (eg, when the user is using the intervention in a public space, sound is turned off)
	22. Adaptiveness to the task	The intervention is aware of the task that the user aims to perform and adapts the interface to this task (eg, the intervention detects the user is making a presentation and the intervention provides the tools generally used, such as inserting text boxes and images)
**Situatedness**		
	23. Prediction of behavior	The intervention accurately predicts the user’s behavior (eg, the intervention detects the user is in the office at 4 p.m. and predicts they will probably go home within the hour)
	24. Adaptation to behavior	The intervention accurately adapts the interface to the predicted behavior (eg, the intervention predicts they will go home within the hour and automatically offers a weather and traffic forecast)
**Social interaction**(these three questions apply to the automated system, and not to contact with a human person such as a therapist or health care professional)		
	25. Embodiment	The online intervention appears humanlike (eg, the intervention has a face, eyes, and/or body that are used for non-verbal communication. This can be in the form of a robot, avatar, or character)
	26. Communication	The intervention applies humanlike communication skills (eg, the intervention expresses social thoughts, feelings, and behaviors)
	27. Following conventions	The intervention follows and applies social conventions (eg, the intervention applies turn taking, cooperation, and/or reciprocity)

### Outcome Measures

Following Webb et al [3], when studies reported the impact of an intervention on multiple outcomes, effect sizes were averaged before inclusion in the main dataset. Where studies included multiple points of measurement, the longest follow-up point was included. Effect sizes were computed as the standardized mean difference between intervention and comparison conditions in study outcomes using Hedges’ *ḡ* correction for small sample size [30]. In our study, the distribution of the effect sizes was dichotomized using the overall effect size as the split point (following the strategy proposed by [19]). The effect sizes of the 52 included interventions ranged from -0.47 to 2.25, with an overall pooled effect size (weighted for sample size) of *ḡ*=0.25 (95% CI 0.18-0.31). The median value was *ḡ*=0.20, which is considered a small effect size, according to Cohen’s criteria [31]. We used this latter value as a criterion for success for this type of intervention. Interventions with an effect size higher or equal to *ḡ*=0.20 were classified as successful (n=26), and those with effect sizes below 0.20 as less successful (n=25).

### Statistical Analysis

Prior to the main analyses, effect sizes were inspected for outliers. The intervention described by Hurling et al [32] appeared to be an outlier (*ḡ*=2.25). Analyses performed with and without this outlier showed that it significantly influenced the weighted average effects and so it was omitted from subsequent analyses.

### Univariate Analyses

To analyze the effect of usability factors on the efficacy of interventions, standard subgroup or moderator analyses, were conducted. The mixed effects model consisted of a random effects model within subgroups and a fixed effect model *across* subgroups, which is an approach recommended by Borenstein et al [33]. The significance of the Q model statistic indicated whether the heterogeneity could be explained by the between groups variable and thus if the factor was a significant moderator [33]. The mixed effects analysis was performed in CMA [29].

### Multivariate Analyses With Meta-CART

Meta-CART consists of two phases. In the first phase, a CART analysis is applied and in the second phase, subgroup meta-analysis is applied to the results of the first phase. CART is a machine learning technique that builds classification trees for categorical outcome variables and regression trees for continuous outcome variables. In the context of our review, the CART algorithm partitions interventions into homogeneous subsets, resulting in a binary tree in which the end nodes contain the most homogeneous groups with respect to within-group effect size. The partitioning is based on intervention characteristics (eg, a BCT). More information on the background of CART analysis is provided by Dusseldorp et al [19].

**Figure 1:**
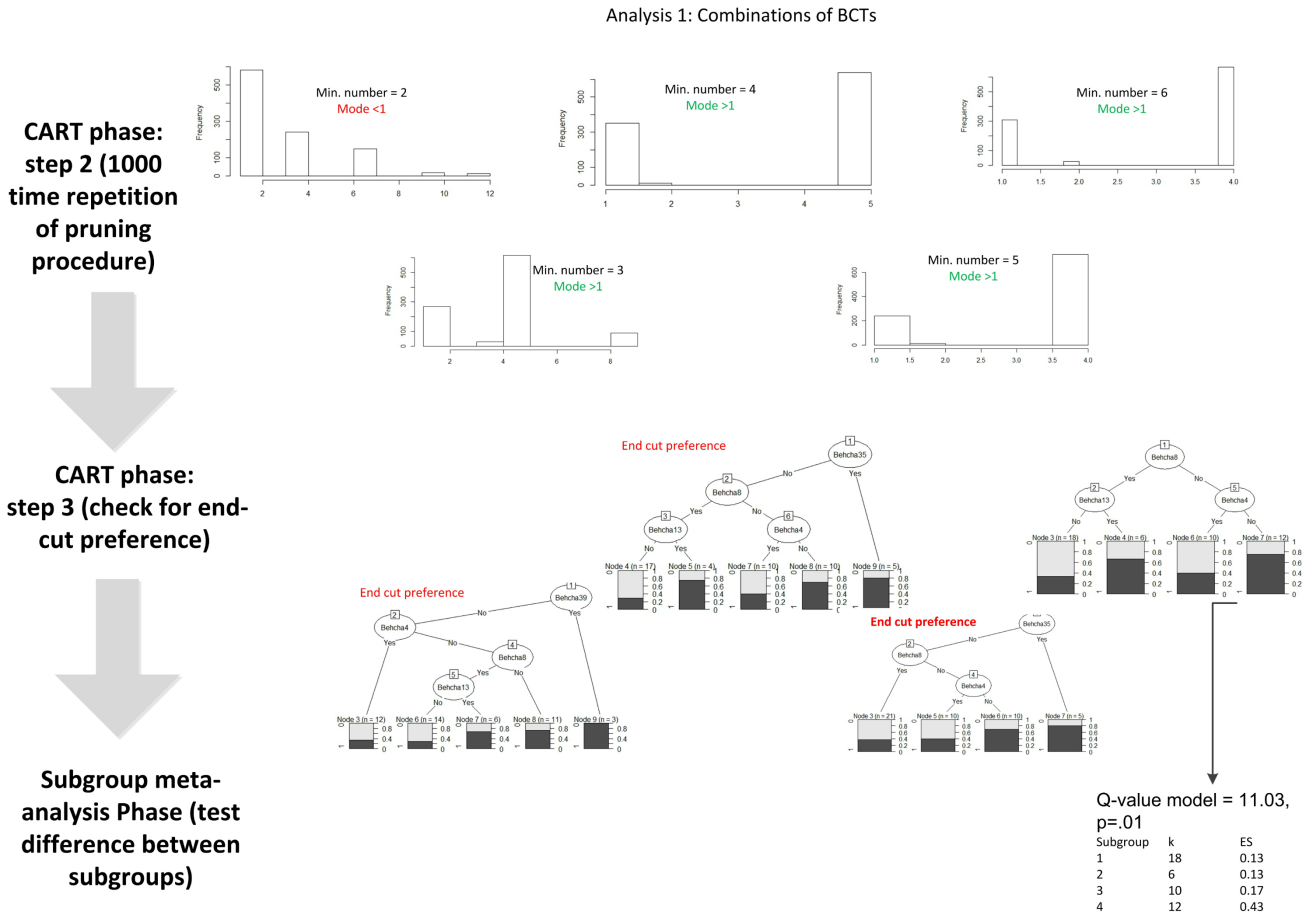
Stepwise meta-CART analyses.

A CART analysis proceeds in three steps (see Figure 1). In the first step, a full classification tree is grown [20] for the dichotomized outcome variable with different minimum numbers of interventions in an end node. This minimum can be fixed at 5 (which is often used in standard CART) or can be varied. In general, the recommended strategy is to choose this value as low as possible (to be able to grow a large tree and then prune it back) [20]. In this study, we varied this number between 2 (the absolute minimum) and 6 (a relatively high value). In addition, a minimal decrease in heterogeneity (impurity) of 0.001 was set as a stopping rule. In this analysis, this resulted in 6 full classification trees that differed in the minimum number of interventions in the end nodes. In the second step, the full classification trees were pruned, using the standard procedure of CART (with tenfold cross-validation and the one-standard-error rule [19]). The pruning procedure results in a best size of the tree, expressed in the number of end nodes. To increase the stability of the results, the pruning procedure was repeated 1000 times [19]. This resulted in 1000 estimates of the best tree size, from which the modal tree size was chosen. If the modal tree size was 1 (meaning a tree of one end node, ie, the total group is not split) or 2 (meaning a tree with two end notes, so one split, indicating no interaction), then the analyses were not continued. For example, in Figure 1, the pruning procedure shows that there is no interaction effect when the minimum number in an end note is set at 2. In the third step, the pruned trees were inspected for “end cut preference” splitting: this occurs if the first split of the tree ends in a node that has the minimum number of interventions (as defined earlier, ranging from 2-6). If this occurs in the first branch, no splits can be made after this one. If end-cut preference was present, the tree was dismissed and a tree with a larger minimum number of interventions was preferred [34]. In this research, end-cut preference occurred when the minimum number was set at 3, 4, and 5. These three steps are shown in Figure 1. The final tree represents the synergistic effect of the moderators (in our case BCTs and or modes) on outcomes. The end nodes of the tree form the subgroups. From the final tree, a new variable was created, with its categories referring to the end nodes of the tree. The CART analyses were performed in the R software environment, version 2.15 [35] using the package rpart, which is developed for classification and regression trees [36].

In the second phase of the meta-CART procedure, a standard subgroup meta-analysis is performed to investigate whether the new grouping variable resulting from the first phase accounts for heterogeneity in the study effect sizes. The same procedures were used as for the analyses of the factors that influence usability. An advantage of the subgroup analysis was that a weighted mean effect size (*ḡ*) was obtained for each subgroup (ie, end node of the tree).

In total, three meta-CART analyses were performed, varying in the BCTs and MoDs that were included as moderators (see Table 2). The first analysis included only the 20 BCTs. The second analysis included only the 7 MoDs. The third analysis included the 20 BCTs and 7 MoDs together. Not enough studies were available to perform a meta-CART analysis with the usability factors as moderators.

**Table 2:** Meta-CART Analyses conducted in this research (see Multimedia Appendix 1 for the names of the BCTs and MoD).

Research question	Included moderators
	BCT/ MoD^a^
Effective combinations of BCTs	Analysis 1: 46 studies
	20 BCTs: 4, 5, 6, 7, 8, 10, 13, 14, 19, 20, 21, 22, 23, 24, 25, 27, 28, 34, 35, 39
Effective combinations of MoD	Analysis 2: 31 studies
	7 MoDs: b, d, e, f, g, h, i
Effective combinations of BCTs and MoDs	Analysis 3: 43 studies
	20 BCTs (see above); 7 MoDs (see above)

^a^BCTs/ MoDs that are univariately associated with an effect size that is higher than the effect size of the studies that do not include the specific BCT/MoD, minus outlier Hurling et al [32].

## Results

First, the scale characteristics and effect sizes from the factors that influence usability are described. Then, the results from the three meta-CART analyses are described: the first one using BCTs only, the second one using MoDs only, and the third one using both.

### Usability and Effect Size

The first analysis examined whether the usability factors influenced intervention effectiveness. Interventions that were deemed to be more efficient (ie, scored higher on the efficiency subscale) proved to be more effective (*ḡ*=0.43, 95% CI 0.18-0.67) than interventions that are less efficient (*ḡ*=0.02, 95%CI -0.29 to 0.10; between groups Q-value=4.25, *P*=.04). No relations between effect size and other usability factors were found (see Table 3).

**Table 3:** Usability: factor reliabilities, factor median, and the impact of these factors on effect size (low versus high, Q-model and *P* value for difference) among 12 interventions.

Scale	α	Median of scale	*ḡ* (95% CI)		Between groups Q (*P* value)
			Low (≤median of scale)	High (>median of scale)	
Learnability^a^	.52^b^	-	-	-	-
Efficiency	.69	3.33	0.02 (-0.29 to 0.10)	0.43 (0.18-0.67)	4.25 (.04)
Memorability	.91	5.00	0.32 (-0.23 to 0.87)	0.22 (-0.09 to 0.53)	0.09 (.76)
Errors^c^	.70	3.33	0.47 (0.31-0.63)	0.15 (-0.34 to 0.64)	1.45 (.23)
Satisfaction^d^	.73	4.00	0.37 (0.01-0.73)	0.15 (-0.22 to 0.53)	0.67 (.41)
Personalization^e^	.70	2.33	0.15 (-0.19 to 0.49)	0.38 (0.05-0.68)	0.82 (.37)
Situatedness	.98	2.00	0.21 (-0.08 to 0.50)	0.52 (0.23-0.81)	2.14 (.14)
Social Interaction	.94	3.33	0.10 (-0.40 to 0.69)	0.34 (0.04-0.65)	0.68 (.41)

^a^Item 4 removed from scale.

^b^Scale not analyzed because of low reliability.

^c^Item 10 removed from scale.

^d^Item 16 removed from scale.

^e^Item 21 removed from scale.

### Effective Combinations of Behavior Change Techniques

**Figure 2:**
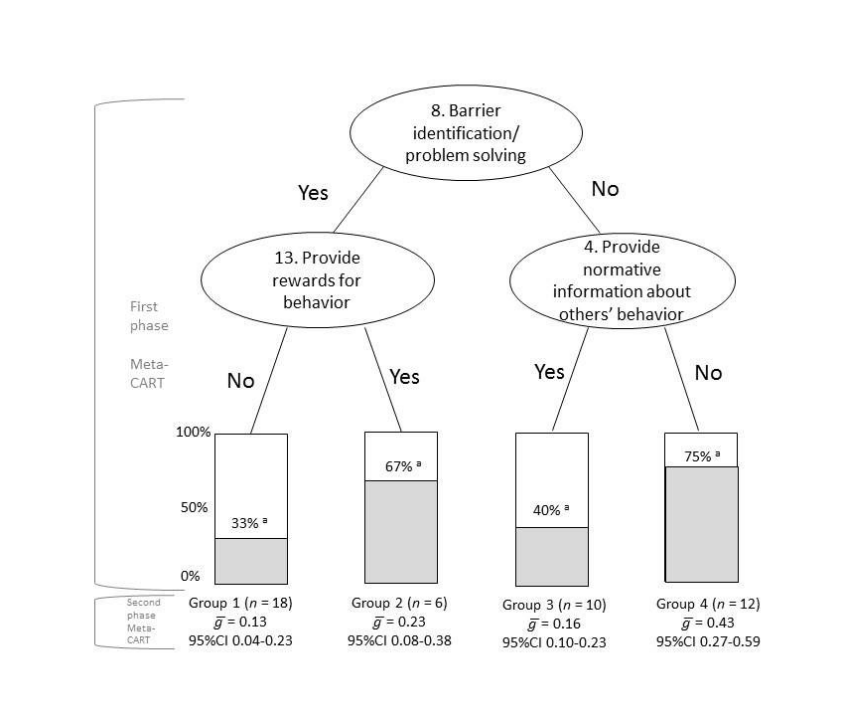
Results from meta-CART and subgroup analyses: classification tree and effect sizes across studies that used at least two univariately effective BCTs (n=47) (note a: percentage of interventions in this node that were more successful, ie, an effect size higher than 0.20).

Meta-CART analyses were conducted to study effective combinations of BCTs. The best fitting tree was found when a BCT was used by at least 6 interventions, that is, the minimum amount of studies in an end node was set at 6. This resulted in a tree with four end nodes (see Figures 1 and 2). Subgroup analysis showed that the subgroups were significantly different from each other (between groups Q-value=11.03, *P*=.01). One synergistic effect was found: interventions that included both barrier identification/ problem solving and provided rewards for behavior change had an average effect size of 0.23 (95% CI 0.08-0.38). Interventions that only provided normative information about the behavior of others (*ḡ*=0.16, 95% CI 0.10-0.23) or included barrier identification/ problem solving (*ḡ*=0.13, 95% CI 0.04-0.23) were least effective. Interventions that used other combinations of techniques than barrier identification/ problem solving and provided rewards for behavior change were most effective (*ḡ*=0.43, 95% CI 0.27-0.59).

### Effective Combinations of Modes of Delivery

The meta-CART analysis including the seven MoDs showed that the best fitting tree had only one node—the root node. In other words, there was no combination of MoDs that was able to explain heterogeneity in the effectiveness of Internet-based interventions for health behavior change.

### Effective Combinations of Behavior Change Techniques and Modes of Delivery

When all BCTs and MoDs were combined in the third analysis, no synergistic effects were found.

### Additional Analyses on Group 4

Figure 2 shows that interventions that did not prompt barrier identification/ problem solving or provide normative information about others’ behavior (Group 4) were the most effective. We therefore conducted additional analyses to understand the positive effects among this group of studies. Based on previous reviews, meta-analyses and the original study by Webb, we set hypothesis related to study quality, intervention intensity, and effective univariate BCTs. Our first hypothesis was that the increased effectiveness was due to the interventions in this group being more intensive. Our second hypothesis was that the interventions with a larger effect were evaluated in studies of lower methodological quality than the interventions with a smaller effect (eg, used passive control conditions or self-report measures of outcome). In order to test these hypotheses, 2 authors coded (1) the number of contacts and length of contact in the intervention, (2) activity in the control condition (no treatment vs alternative intervention), and (3) measurement of outcome (self-report or objective). They discussed the coding results until agreement was reached.

Next, we compared the BCTs, intervention, and characteristics from the studies in Group 4 (n=12) to the other studies in this review (n=34), using chi-square tests and *t* tests. Interventions in Group 4 had significantly more contact moments (mean=180) than the other interventions (mean 40; *P*=.03). Furthermore, the BCTs “model/demonstrate the behavior” (*P*=.09) and “prompt self-talk” (*P*=.01) were more often used in Group 4 studies compared to the other studies. These BCTs were not included in the meta-CART because they were used by fewer than 6 studies. The interventions in Group 4 also used an active control intervention more often (compared to no intervention, *P*=.03) and had a longer follow-up time between intervention and measurement (*P* <.05) than the other studies. No differences were found with regard to use of self-reporting or objective measurement of outcomes. Thus, the higher effects in this group cannot be explained by a weaker study design in these studies.

## Discussion

### Principal Findings

This meta-analysis re-analyzed data from a systematic review of online interventions aimed at health-related behavior change [3] in an effort to identify synergistic effects of behavior change techniques (BCTs), modes of delivery (MoDs), and usability factors. First, the univariate analyses of the usability factors indicated that one usability factor influenced effect sizes: the efficiency of the intervention. Meta-CART was then used to identify subgroups of interventions that were associated with particular levels of effectiveness. One synergistic effect was found: barrier identification/ problem solving in combination with providing rewards for behavior change (*ḡ*=0.23). No synergistic effects were found among MoDs or among MoDs with BCTs. Below, we discuss each of these findings in more detail.

### Usability Factors

The usability questionnaire that we developed resulted in 7 reliable subscales (one subscale was not reliable). Efficiency was positively related to intervention effect. This means that interventions that are flexible (can cater both experienced and inexperienced users), provide structure (that is understood by the user and the user knows where they are in that structure), and make use of default settings are more likely to be effective. The score for efficiency ranged from 2.33 (for study [37]) to 5 (for study [38,39]). Participants may be more likely to use an intervention if it is efficient in its use (eg, quick delivery of information or support). Furthermore, these characteristics allow the user to use the system easily and quickly, therefore posing low cognitive load on the user. A high cognitive load is less likely to result in a learning experience than a low cognitive load [40]. Based on these assumptions, increasing the content of an online intervention may come at the expense of efficiency. This makes it necessary to take into account usability and effectiveness when making a strategic choice about what to include in interventions.

Nevertheless, we should be careful in interpreting these results because usability information was provided by the authors of only 12 of the primary studies and their answers were not cross-validated as we did not have access to these interventions. However, based on the reliability of the subscales (the majority) and the finding that efficiency predicted variability in the effectiveness of the primary interventions, we believe that our taxonomy is suitable for use in these kinds of settings. Of course, more research in larger studies is needed to establish its reliability and validity, especially if coded by different authors. A special concern in these studies should be the improvement of the learnability scale, as its reliability was unsatisfactory. Better-powered studies should explore the best combinations of items to make the scales. In addition, item 4 (visibility of system status) was removed because it reduced the reliability of the scale even further. The remaining three items have a mean value of 4.4-4.6 (SD 0.70-0.97). As the minimum is 1 and the maximum is 5, this may indicate that these questions cannot differentiate sufficiently. Perhaps the questions should be stated in a stronger and more precise fashion. For example, instead of asking if the intervention follows platform conventions (item 2; scale of totally disagree to totally agree), it may be possible to ask which or how many aspect platform conventions are followed (eg, cross in upper right corner, disk to save changes).

### Synergistic Effects

Webb et al [3] reported that the univariate effects of barrier identification/ problem solving and providing rewards for behavior change were *ḡ*=0.20 and *ḡ*=0.18, respectively. In this analysis, these two BCTs had a combined effect of *ḡ*=0.23, which is higher than using barrier identification/ problem solving only. Both techniques are likely to play a role in the maintenance of action and prevention of relapse. The interactive effect may be due to their different approach. While barrier identification/ problem solving takes a somewhat negative approach (ie, thinking about what can go wrong), providing rewards for behavior change suggests a positive consequence in the future. Also, barrier identification/ problem solving is aimed directly at problem solving (instrumental function of skill learning), while providing rewards for behavior change has a more affective function in increasing motivation. The combination may thus provide for different needs. Based on our findings, we suggest using these techniques together.

However, the most effective interventions were those that did not provide normative information about the behavior of others or prompt barrier identification/ problem solving (*ḡ*=0.43). Webb et al [3] showed that interventions including more BCTs were more effective, but this group of studies did not use more BCTs than the less effective studies. However, the effective studies had significantly more contact moments than the other studies. In addition, these studies more often used “model/demonstrate the behavior” and “prompt self-talk” than the other studies. Furthermore, it is likely that these studies (and also the other studies) included BCTs that were not included in the taxonomy. Thus, they are not coded and their effects not analyzed by Webb, nor by us in this study. Also, implementation of the BCTs was not taken into account, a factor that may influence effectiveness [41] by how, when, and for how long a BCT is used in an intervention. For example, the BCT “provide rewards for behavior” does not make explicit what the reward is, who decides it, and how often it is given. These factors can greatly influence its effectiveness.

In line with previous studies, our findings suggest that online interventions designed to promote health behavior should not provide normative information on the behavior of others. Other studies showed that providing information on what people usually do (ie, descriptive norms), independent of presenting minority or majority information [42], is less likely to result in action [39,43,44]. When presenting normative information, it might be better to use injunctive norms (what people typically approve or disapprove) [45]. In future reviews it will be important to differentiate between injunctive and descriptive norms and then analyze differential effects.

It is important to understand the context in which a BCT operates [42-44]. MoD is part of this context. Michie et al [18] showed that differences in the effectiveness of the interventions could be explained by, among others, theoretical basis, use of theory and MoD. In the original review [3], the univariate influence of additional MoD was quite strong (eg, Internet-based interventions that also included telephone contact were more effective than those without). In our research, however, no effective combinations were found among MoD. This may be due to the fact that each MoD by itself is already an additional mode: every mode is in addition to an online intervention. Based on the results of Webb et al’s review and our meta-analysis, we can recommend that online interventions include one or more additional MoDs, but we cannot advise on specific additional modes to increase the effectiveness. In addition, when BCTs and MoDs were combined, only combinations of BCTs were found. These findings indicate that the effectiveness of BCTs is not dependent on (additional) modes of delivery.

### Limitations and Future Directions

This is one of the first studies to investigate interactive effects of BCTs and MoDs in online interventions. However, the lack of primary studies that used a large number of BCTs and MoDs limited our options for analysis. First, not all BCTs from the original taxonomy of Abraham and Michie [9] were present in the original analyses by Webb et al [3]. Furthermore, only a selection of the BCTs from Webb et al was included in the meta-CART analysis. In addition, other techniques that are not part of the taxonomy (such as cognitive restructuring) may be effective as well. A similar limitation occurred for modes of delivery. As such, our findings are limited to the relatively small number of effective BCTs and MODs that were present in at least 6 interventions (the number needed in each end node to obtain a stable tree) in this dataset. Stress management, general communication skills training, model/demonstrate the behavior, and facilitate social comparison were highly effective univariately but were used by five studies or fewer. As such, these BCTs were not included in the final tree. Thus, the lack of effects found for these BCTs in this meta-analysis does not influence the conclusions about these BCTs in [3]. Furthermore, all studies in the meta-analysis were published at least 7 years ago. As such, no interventions using smartphones or tablets have been included. It would be interesting to study if the same effects are found by more recent interventions, and if the same relation with usability exists.

In general, few synergistic effects were found, suggesting that the BCTs that were identified by Webb et al [3] have a clear single effect that cannot [46] be strengthened by combining them with other BCTs. For example, previous studies have shown that self-monitoring of behavioral outcomes is moderately-to-strongly related to behavior change [3,18,47-50]. In the current meta-analysis, we could not identify other BCTs that improve the effects of self-monitoring. The lack of this combined effect may be due to the same reason why we did not find any combination among some of the most effective BCTs: the low number of studies that used a certain BCT. Another problem may be the dichotomization of the effect size (using a classification tree). Ideally, the original, continuous outcome would be used (in a regression tree); however, the studies could not be weighted. In the CART procedure, the accuracy of the study effect sizes could not be taken into account. The advantage of the use of a continuous outcome (compared to a dichotomized one) is that the variance in the effect sizes is maintained. Therefore, a regression tree is less likely to be biased than a classification tree. The next step for CART in meta-analysis is to develop the possibility of taking study weight into account, thus decreasing the risk of bias in trees.

This meta-analysis aimed to provide information on which combination of techniques may enhance the effectiveness of Internet-based interventions designed to promote health behavior. It does not, however, explain how the techniques should be used in practice, that is, when, where, and in which shape they will be effective. Bartholomew et al provide such recommendations for a limited set of techniques [51], but more information is needed. Also, negative effects were not explored further in the meta-CART analyses because interactions were found only when factors that have positive effects were included. Another limitation is that this meta-analysis shows the effects on behavior and/or outcomes, but not on the potential mediators (eg, factors such as attitudes, self-efficacy, and skills) that might explain behavior change [46,52]. Given that behavior change is assumed to be the result of changes via behavioral or environmental determinants [21,53], and BCTs are expected to have an impact on such determinants, it is important to also understand the change mechanisms [54]. More evidence is also needed with regard to implementation factors such as dose and fidelity [13,55,56]. A higher number of intended contact moments was associated with a larger effects size in the Group 4 studies, but we did not take into account the intended or delivered dose of BCTs. Adding a measure of actual dose allows us to study a dose-effect relation and differentiate between efficacy and effectiveness [41] and may thus increase our understanding of when and how to choose and use BCTs.

Further research into usability factors may also increase our insight in change mechanisms. The lists of indicators of usability and mode of delivery that were used in this study are not exhaustive. Future studies should take into account recent developments in this field, such as the use of smartphones and their connection with the intervention under study. Finally, a broad range of outcomes was used in this meta-analysis (physical activity, healthy eating, alcohol consumption and more) and the relatively small number of studies did not allow us to conduct separate analyses for each behavior. In future studies, it would be useful to find out if combinations of BCTs have different effects for specific behaviors.

### Conclusion

This research developed a new coding frame for identifying the factors that influence the usability of online interventions. One factor—efficiency—influenced effect sizes, with more efficient studies tending to report larger effects than less efficient studies. We then used meta-CART analysis to investigate the effects of combinations of BCTs and/or modes of delivery. We were able to identify the synergistic effect of two BCTs: (1) prompting barrier identification/ problem solving in combination with (2) providing rewards for behavior change. However, no other interactive effects of BCTs and modes of delivery were found.

## Appendix

Multimedia Appendix 1 Univariate effect sizes of behavior change techniques and modes of delivery (n=85 studies).

## Abbreviations

BCT: behavior change technique
CALO-RE: Coventry, Aberdeen, and London—Refined taxonomy
CART: classification and regression trees
CMA: comprehensive meta-analysis
MoD: mode of delivery
