# Supplementary material for: Which Combinations of Techniques and Modes of Delivery in Internet-Based Interventions Effectively Change Health Behavior? A Meta-Analysis
Source: J Med Internet Res. 2016 Jun 7;18(6):e155. doi: 10.2196/jmir.4218 (PMC4936795; doi:10.2196/jmir.4218)
Supplement: Multimedia Appendix 1 [file jmir_v5i2e61_app1.pdf]

Appendix 1 Univariate effect sizes

Effect sizes of behavior change techniques and modes of delivery (n=85 studies).

Behavior Change Technique	Factor included			Factor not included		Included in analyses?		
	k	ES	95% CI	ES	95% CI	Q-value	p	
25. Prompt practice	2	**0.66**	0.39-0.93	**0.15**	0.08-0.22	12.81	0.00	In analysis 1&3 [a, c]
35. Stress management	5	**0.50**	0.27-0.72	**0.14**	0.08-0.21	8.35	0.00	In analysis 1&3 [a]
39. General communication skills training	3	**0.49**	0.25-0.73	**0.15**	0.08-0.21	7.48	0.01	In analysis 1&3 [a]
23. Environmental restructuring	2	**0.45**	0.03-0.12	**0.16**	0.09-0.22	2.97	0.09	In analysis 1&3 [a, c]
21. Model/demonstrate the behavior	5	**0.35**	-0.01-0.70	**0.15**	0.08-0.22	1.19	0.28	In analysis 1&3 [a]
6. Goal setting (outcome)	2	**0.33**	-0.22-0.89	**0.16**	0.09-0.22	0.39	0.53	In analysis 1&3 [a, c]
34. Relapse prevention/coping	14	**0.32**	0.17-0.47	**0.13**	0.06-0.20	4.68	0.03	In analysis 1&3

planning								
27. Facilitate social comparison	4	**0.29**	0.04-0.55	**0.16**	0.09-0.22	1.04	0.31	In analysis 1&3 [a]
5. Goal setting (behavior)	25	**0.27**	0.16-0.38	**0.11**	0.04-0.19	5.19	0.02	In analysis 1&3
7. Action planning	18	**0.25**	0.13-0.37	**0.13**	0.05-0.21	2.92	0.09	In analysis 1&3
19. Provide feedback on performance	19	**0.22**	0.09-0.34	**0.14**	0.07-0.22	0.95	0.33	In analysis 1&3
8. Barrier identification /problem solving	26	**0.20**	0.10-0.30	**0.14**	0.06-0.22	0.90	0.34	In analysis 1&3
20. Provide instruction	25	**0.20**	0.13-0.28	**0.14**	0.05-0.23	1.08	0.30	In analysis 1&3
22. Teach to use prompts/cue s	3	**0.20**	-0.17-0.57	**0.16**	0.09-0.23	0.05	0.83	In analysis 1&3 [a]
4. Provide normative information about others' behavior	16	**0.18**	0.07-0.28	**0.15**	0.07-0.23	0.18	0.67	In analysis 1&3

28. Plan social support/social change	15	**0.18**	0.10-0.27	**0.15**	0.08-0.23	0.28	0.60	In analysis 1&3
13. Provide rewards for behavior	7	**0.18**	0.09-0.28	**0.15**	0.09-0.22	0.26	0.61	In analysis 1&3
10. Prompt review of behavioral goals	2	**0.17**	0.01-0.22	**0.16**	0.09-0.23	0.02	0.88	In analysis 1&3 [a, c]
14. Shaping (contingent rewards)	2	**0.17**	0.01-0.34	**0.16**	0.09-0.23	0.02	0.88	In analysis 1&3 [a, c]
16. Prompt self-monitoring of behavior	28	**0.16**	0.07-0.24	**0.16**	0.07-0.24	0.00	0.99	No, in none [b]
1. Provide information on the consequences in general	29	**0.14**	0.06-0.21	**0.17**	0.07-0.26	0.23	0.63	No, in none [b]
2. Provide information on the consequences for individual	12	**0.14**	0.04-0.24	**0.17**	0.09-0.24	0.21	0.65	No, in none [b]

26. Use of follow up prompts	5	**0.13**	-0.10-0.35	**0.16**	0.09-0.23	0.09	0.77	No, in none [b]
17. Prompt self-monitoring of behavioral outcome	13	**0.12**	-0.03-0.26	**0.17**	0.10-0.24	0.34	0.56	No, in none [b]
12. Reinforcing effort toward behavior	3	**0.11**	0.02-0.19	**0.17**	0.10-0.24	1.08	0.30	No, in none [b]
36. Emotional control training	11	**0.09**	-0.03-0.22	**0.17**	0.10-0.25	1.11	0.29	No, in none [b]
3. Provide information about others' approval	5	**0.06**	-0.11-0.23	**0.17**	0.10-0.24	1.46	0.23	No, in none [b]
24. Agree behavioral contract	1							No, in none [c]
31. Fear Arousal	1							No, in none [c]
32. Prompt self-talk	1							No, in none [c]
37. Motivational	1							No, in none [c]

interviewing		
9. Set graded tasks	0	No, in none [c]
11. Prompt review of outcome goals	0	No, in none [c]
15. Prompting generalization of behavior	0	No, in none [c]
18. Prompting focus on past success	0	No, in none [c]
29. Prompt identification as role model	0	No, in none [c]
30. Prompt anticipated regret	0	No, in none [c]
33. Prompt use of imagery	0	No, in none [c]
38. Time managemen	0	No, in none [c]

t										
40. Provide non-specific social support	0									No, in none [c]
	**Mode of Delivery**									
9. Text message (i in Webb et al, 2010)	4	**0.81**	0.14-1.49		**0.14**	0.07-0.20		3.82	0.05	Analyses 2&3
12. Telephone (h)	7	**0.35**	0.09-0.61		**0.15**	0.08-0.22		2.11	0.15	Analyses 2&3
7. Access to advisor to request advice (d)	23	**0.29**	0.16-0.42		**0.12**	0.04-0.19		5.04	0.03	Analyses 2&3
6. Scheduled contact with advisor (e)	13	**0.22**	0.09-0.36		**0.15**	0.07-0.21		0.99	0.32	Analyses 2&3
8. Peer to peer access (f)	20	**0.20**	0.09-0.31		**0.15**	0.07-0.23		0.52	0.47	Analyses 2&3
4. Automated tailored	18	**0.18**	0.07-0.28		**0.16**	0.08-0.24		0.07	0.79	Analyses 2&3

feedback (b)								
13. Email (g)	19	**0.18**	0.07-0.29	**0.15**	0.07-0.23	0.16	0.69	Analyses 2&3
5. Enriched information environment (a)	30	**0.15**	0.07-0.23	**0.16**	0.07-0.25	0.03	0.86	No, in none [b]
2. Automated follow-up messages (c)	14	**0.09**	-0.01-.19	**0.17**	0.10-0.25	1.55	0.21	No, in none [b]
11. CD-rom (j)	1							No, in none [c]
14. Video conferencing (k)	1							No, in none [c]

[a] Not in final model because Behavior Change Technique /Mode of Delivery is applied by less than 6 studies
[b] *g* for using this Behavior Change Technique /Mode of Delivery is similar or smaller than not using that Behavior Change Technique /Mode of Delivery
[c] Not tested in original meta-analyses (Webb, 2010).
